# Use of sedation‐awakening electroencephalography in dogs with epilepsy

**DOI:** 10.1111/jvim.17153

**Published:** 2024-08-12

**Authors:** Marcin Wrzosek, Aleksandra Banasik, Adriana Czerwik, Agnieszka Olszewska, Marta Płonek, Veronika Stein

**Affiliations:** ^1^ Department of Internal Diseases with a Clinic for Horses, Dogs and Cats, Faculty of Veterinary Medicine Wrocław University of Environmental and Life Sciences Wrocław Poland; ^2^ NeuroTeam Specialist Veterinary Clinic Wrocław Poland; ^3^ Department of Veterinary Clinical Sciences, Small Animal Clinic Justus‐Liebig‐University Giessen Germany; ^4^ Evidensia Small Animal Hospital Arnhem The Netherlands; ^5^ Division of Clinical Neurology, Department for Clinical Veterinary Medicine, Vetsuisse Faculty University of Bern Bern Switzerland

**Keywords:** ambulatory EEG, canine epilepsy diagnosis, canine electroencephalography, epilepsy, paroxysmal, sedation, seizure

## Abstract

**Background:**

Electroencephalography (EEG) recording protocols have been standardized for humans. Although the utilization of techniques in veterinary medicine is increasing, a standard protocol has not yet been established.

**Hypothesis:**

Assessment of a sedation‐awakening EEG protocol in dogs.

**Animals:**

Electroencephalography examination was performed in a research colony of 6 nonepileptic dogs (control [C]) and 12 dogs with epilepsy admitted to the clinic because of the epileptic seizures.

**Methods:**

It was a prospective study with retrospective control. Dogs with epilepsy were divided into 2 equal groups, wherein EEG acquisition was performed using a “sedation” protocol (IE‐S, n = 6) and a “sedation‐awakening” protocol (IE‐SA, n = 6). All animals were sedated using medetomidine. In IE‐SA group, sedation was reversed 5 minutes after commencing the EEG recording by injecting atipamezole IM. Type of background activity (BGA) and presence of EEG‐defined epileptiform discharges (EDs) were evaluated blindly. Statistical significance was set at P > 0.05.

**Results:**

Epileptiform discharges were found in 1 of 6 of the dogs in group C, 4 of 6 of the dogs in IE‐S group, and 5 of 6 of the dogs in IE‐SA group. A significantly greater number of EDs (spikes, *P* = .0109; polyspikes, *P* = .0109; sharp waves, *P* = .01) were detected in Phase 2 in animals subjected to the “sedation‐awakening” protocol, whereas there was no statistically significant greater number of discharges in sedated animals.

**Conclusions and Clinical Importance:**

A “sedation‐awakening” EEG protocol could be of value for ambulatory use if repeated EEG recordings and monitoring of epilepsy in dogs is needed.

**Abbreviations**
AEEG: ambulatory wireless video electroencephalography
ASD: antiseizure drugs
BGA: background activity
C: control
EEG: electroencephalography
EDs: epileptiform discharges
EMG: electromyographic
HVLF: high voltage, low frequency
IE‐S: epileptic‐sedation
IE‐SA: epileptic‐sedation‐awaking
ILAE: International League Against Epilepsy
IVETF: International Veterinary Epilepsy Task Force
LVHF: low voltage, high frequency
NREM: nonrapid eye movement
REM: rapid eye movement
SITs: superimposed transients
SNE: subdermal needle electrodes
SWE: subdermal wire electrodes
SWS: slow wave sleep

## 1 INTRODUCTION

Electroencephalography (EEG) is a basic diagnostic method used for assessment of dogs with epilepsy. This diagnostic technique allows for the definition, classification, and advanced understanding of the pathophysiology of the disease and improves the therapeutic management plan for epilepsy disorders in veterinary medicine, similar to human medicine.^1^, ^2^, ^3^, ^4^ Electroencephalography allows the identification and documentation of paroxysmal discharges as subclinical events without observing an actual seizure in an animal. This plays a crucial role in ascertaining the diagnosis of epilepsy and potentially defines a type of epilepsy as a specific therapy type. An accurate definition of a phenotype must include EEG data obtained and interpreted by a trained neurologist specialized in investigation of epileptic disorders, especially for dogs with a suspected genetic etiology.^5^, ^6^

Although the International Veterinary Epilepsy Task Force (IVETF) recommends the use of EEG in diagnosing seizure disorders in dogs, it is still underutilized in canine medicine and this diagnostic method has not been standardized by the IVETF.^4^, ^7^ This could be a barrier to precisely classify seizure origin, accurately define seizure type in dogs, and potentially inhibit the therapeutic options for this species. The main challenge in this field is the technical difficulty of obtaining valuable readable EEG records.

Electroencephalography acquisition in dogs is performed using various electrodes placed on the skin surface as cups or in the form of needles.^7^, ^8^, ^9^, ^10^, ^11^, ^12^, ^13^, ^14^ The proper technical and adequate qualitative recording in dogs was reportedly done using needle electrodes, such as subdermal needle electrodes (SNE) or subdermal wire electrodes (SWE).^12^, ^15^ Appropriate placement of needle electrodes without sedation might be stressful for the animal. Therefore, it can be technically difficult to perform EEG in some cases and could have a high potential for several movement artifacts. Most reported EEG recordings with a use of needle electrodes in dogs have been performed under sedation, with or without use of a reversal agent. A common reason for sedation is the ability to install electrodes properly. However, sedation can alter the EEG recordings in dogs.^15^, ^16^, ^17^ Sedatives and anesthetics change the EEG picture, which makes it difficult to interpret and compare the records in dogs. Literature on EEG in dogs does not provide information on the effects of sedatives and sedative antagonists on the quantity and quality of epileptiform discharges (EDs) in EEG recordings. Therefore, a practical, easy‐to‐use EEG recording protocol for application in routine ambulatory dogs that facilitates the installation of electrodes and yields maximum reliable recordings should be formulated.

This study had 2 aims: (1) qualitative and technical analysis of the “sedation‐awake” protocol for performing short‐term ambulatory EEG recordings in dogs; (2) to assess the EEG “sedation‐awakening” protocol in terms of the observed background activity (BGA), EDs, and possible artifacts in a 30‐minute recording and compare it with control group and standard EEG protocol previously described.

## 2 MATERIALS AND METHODS

A prospective EEG acquisition was performed on 12 epileptic dogs that were admitted for neurological consultation at the Department of Internal Medicine and Clinic of Diseases for Horses, Dogs and Cats, Faculty of Veterinary Medicine, Wroclaw University of Environmental and Life Sciences between January 2015 and June 2022. Previously reported normal nonepileptic dogs from the clinical database were included in the control group. Information retrieved from the clinical database included the clinical, neurological, and EEG findings of the dogs.^15^ In accordance with the Polish law, this study did not require the approval of the Ethical Committee (Experiments on Animals Act from January 15, 77 2015, Journal of Laws of the Republic of Poland from 2015, item. 266).

### 2.1 Subjects

The inclusion criteria for the study group were as follows: age between 6 months and 6 years at seizure onset (all dogs were diagnosed with generalized tonic‐clonic seizures), history of at least 2 epileptic seizures, a minimum time period 16 weeks from the epileptic seizure to the performance of the EEG examination, the quality of the EEG recording that allows its evaluation (small amount of motion artifacts visible on video, clear signal from electrodes), normal interictal clinical and neurological examination, blood analysis results without any abnormalities (complete blood count, biochemistry including bile acids, and ammonia), no abnormalities detected on MRI examination performed according to the IVETF protocol and no abnormalities detected in cerebrospinal fluid analysis (CSF) (consistent with IVETF Tier II).^4^ The exclusion criterion was a history of cluster seizures or status epilepticus to avoid the influence of severity of seizures on the number of EDs.

The inclusion criteria for the control group were as follows: no history of seizures or any other forebrain disease, normal clinical and neurological examination results, normal blood measurements, normal brain MRI findings.

The dogs were divided into the following 3 groups: group C (control), nonepileptic dogs with a medetomidine sedation protocol recording; group IE‐S (epileptic‐sedation), dogs with idiopathic epilepsy with an EEG recorded under the same standard medetomidine sedation protocol; and group IE‐SA (epileptic‐sedation‐awakening), dogs with idiopathic epilepsy and an EEG acquisition using the “sedation‐awakening” protocol. Dogs with IE were randomly assigned to the IE‐S or IE‐SA group after qualifying for EEG.

### 2.2 EEG recording

**FIGURE 1:**
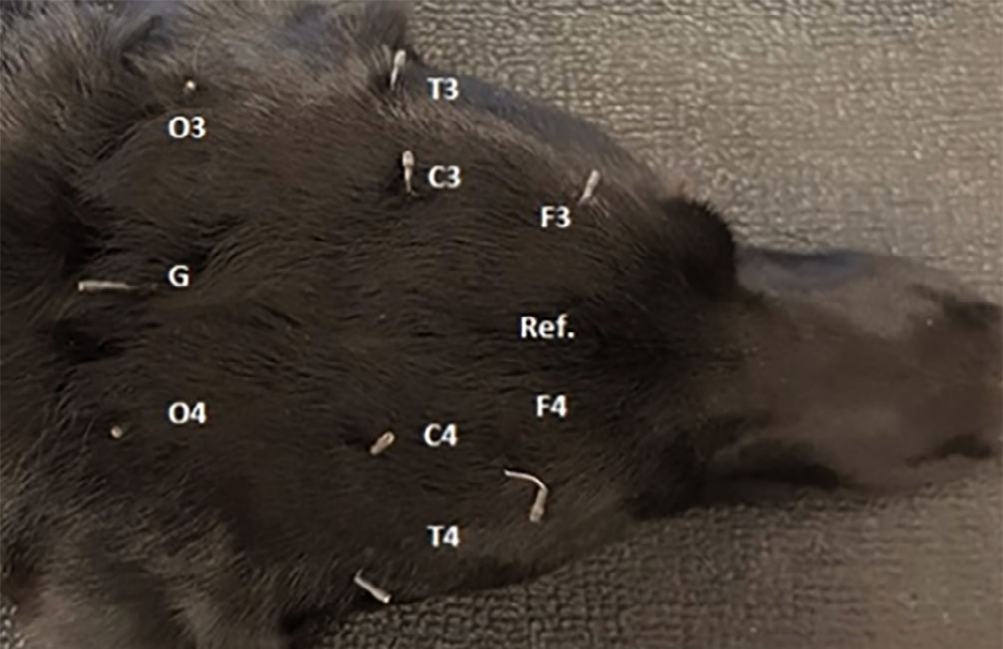
SWE montage. SWE, subdermal wire electrodes.

Dogs were sedated using IM injections of medetomidine. The drug was injected into the right triceps muscle at a dose of 20 μg/kg and an additional 10 μg/kg was administered, if the dog was not ready for manipulation 15 minutes after the initial injection. The recordings were obtained using SWE (Figure 1). To prevent eventual electrode displacement after the animals awoke, the electrodes were fixed on the head of each dog in the IE‐SA group using an elastic bandage (Figure 1). In groups C and IE‐S, the assumed short‐term recordings lasted for a minimum of 30 minutes.

In the IE‐SA group, sedation was reversed 5 minutes after commencing the EEG recording by injecting atipamezole intramuscularly (40 μg/kg body weight) to continue the recording after the reversal of the sedative.

**FIGURE 2:**
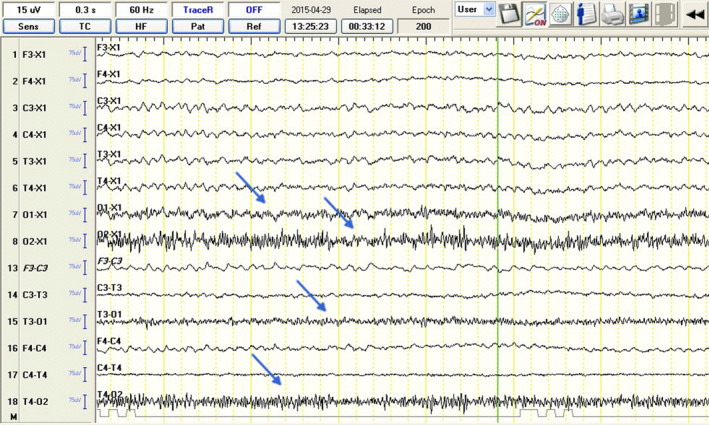
Electroencephalographic recordings from a 4‐year‐old male cavalier King Charles spaniel showing muscular activity artifacts in O1, O2 derivations (blue arrows).

The recordings were carried out using an EEG unit with the following settings: sensitivity, 70 μV/cm; band pass filter, 0.5 to 30 Hz; time constant, 0.3 second, and an inserted 50 Hz notch filter. Each recording was performed using an 8‐channel referential montage (F3, F4, C3, C4, T3, T4, O1, O2; Ref., the reference electrode was placed on the frontal bone, the ground electrode was inserted in the neck and in a standard bipolar montage [F3‐C3, C3‐T3, T3‐O1, F4‐C4, C4‐T4, T4‐O2]). The electrocardiogram reference (ECG‐Ref.) electrode was placed SC at the level of the left fifth intercostal space near the chondrocostal junction. In groups C and IE‐S, light stimulation using a stroboscope lamp was conducted in the fifth recording minute. In the IE‐SA group, stimulation was administered immediately after the administration of atipamezole. The initial stimulation frequency was 0.5 Hz, which was gradually increased to 60 Hz, and then steadily decreased to the base point over a 5 minutes period, as previously reported.^15^ All recordings were studied blindly by a veterinary neurologist (blinded for revision) using monopolar and simultaneous bipolar montage and videometric control (Figure 2). Qualitative analysis (evaluation of BGA, number and type of EDs) in all 3 groups was done and technical analysis (description of the study, difficulties in conducting, and assessing the study) of the “sedation‐awake” protocol was performed and compared with IE‐S and C group.

**FIGURE 3:**
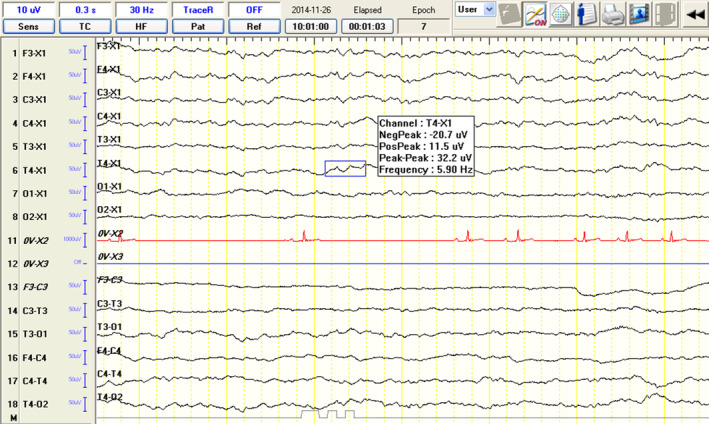
Electroencephalographic recordings from a 6‐year‐old male Siberian husky showing high voltage, low frequency (HVLF) background activity.

**FIGURE 4:**
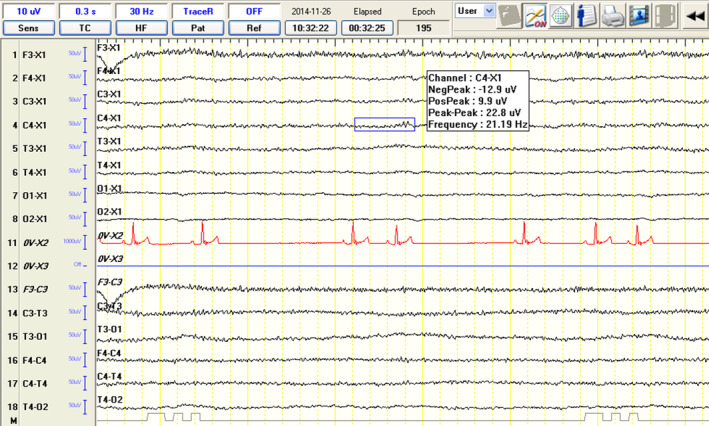
Electroencephalographic recordings from a 6‐year‐old male Siberian husky showing low voltage, high frequency (LVHF) background activity.

Background activity was defined based on visual assessment and frequency measurement. Background activity was defined as low voltage, high frequency (LVHF), if the frequency exceeded 8 Hz and amplitude ranged between 10 and 40 μV (Figure 3). On the other hand, BGA was classified as high voltage, low frequency (HVLF) if the frequency ranged between 4 and 7 Hz and amplitude between 40 and 140 μV (Figure 4). Physiological and pathological superimposed transients (SITs) along with motion and electromyographic (EMG) artifacts were recorded on a transient annotation list. Attention was paid to differentiating the SITs based on movement and environmental artifacts.

The currently accepted nomenclature was used to define EDs.^8^, ^18^ A spike was defined as “a transient wave, clearly distinguished from the BGA, with a pointed peak at conventional paper speed and duration ranging between 20 and <70 ms; with a generally negative main component.” A sharp wave was defined as “a transient wave, clearly distinguished from the BGA, with a pointed peak at conventional paper speed and duration ranging between 70 and 200 ms; with the main component being generally negative,” and a spike wave‐complex was defined as “a pattern consisting of a spike followed by a slow wave.” Polyspikes and polysharp waves were combinations of multiple spikes and sharp waves. Epileptiform discharges localization was defined based on the highest amplitude of discharge in a reference montage and reversed polarity in the bipolar montage recording. The results were compared blindly between the protocols and groups.

### 2.3 Statistical analysis

Nonparametric analysis of variance for repeated measures was used to compare changes over time in variables including the 3 groups involved in the study. For post hoc comparisons, a multiple comparisons test was used. The level of statistical significance was set at *P* < .05, the analysis was performed in R in the Rstudio environment by the tidyverse, ggplot2, and prcomp packages.

## 3 RESULTS

In this study, we included 6 dogs each in the IE‐S, IE‐SA, and C groups. The IE‐S group consisted of 5 female and 1 male dog, aged 24 to 60 months (median, 38 months) and weighing between 6 and 30 kg (median, 19.8 kg). The IE‐S group included 2 mixed breeds, 2 German shepherds, and 1 each of border collie and Akita Inu breeds. The IE‐SA group comprised 5 male and 1 female dog, aged 24 to 72 months (median, 42 months) and weighing between 10 and 35 kg (median, 18 kg). The IE‐SA group included 2 mixed breeds and 1 each of Siberian husky, Labrador retriever, border collie, and beagle breeds. Group C consisted of 2 male and 4 female dogs, aged 48 to 108 months (median, 61.5 months) and weighing between 7 and 30 kg (median, 17.5 kg). The C group included 2 mixed breeds and 4 beagles.

### 3.1 EEG recording

To visually analyze the EEG recordings, they were divided into 2 parts: Phase 1, the first 5 minutes of recording under medetomidine sedation; and Phase 2, which commenced from the fifth minute (atipamezole injection—atipamezole injection in IE‐SA group) and lasted until total recovery from sedation (eyes open, dogs started to move) or up to the 30th minute of recording. The results of visual analysis are listed in Table 1. The initial recording observed in still sedated animals was slow wave sleep (SWS), which was determined by the presence of HVLF BGA along with vertex sharp waves, sleep spindles, and K‐complexes.

**TABLE 1:** Results of visual analysis.

	Phase 1			Phase 2		
	BGA	Physiological SIT	EDs	BGA	Physiological SIT	EDs
Group C	HVLF	28	3	HVLF	15	3
Group IE‐S	HVLF	24	46	HVLF	40	43
Group IE‐SA	HVLF	13	71	LVHF	11	114

Abbreviations: BGA, background activity; C, control; EDs, epileptiform discharges; HVLF, high voltage low frequency; IE‐S, epileptic‐sedation; IE‐SA, epileptic‐sedation‐awaking; LVHF, low voltage high frequency; SIT, superimposed transients.

**FIGURE 5:**
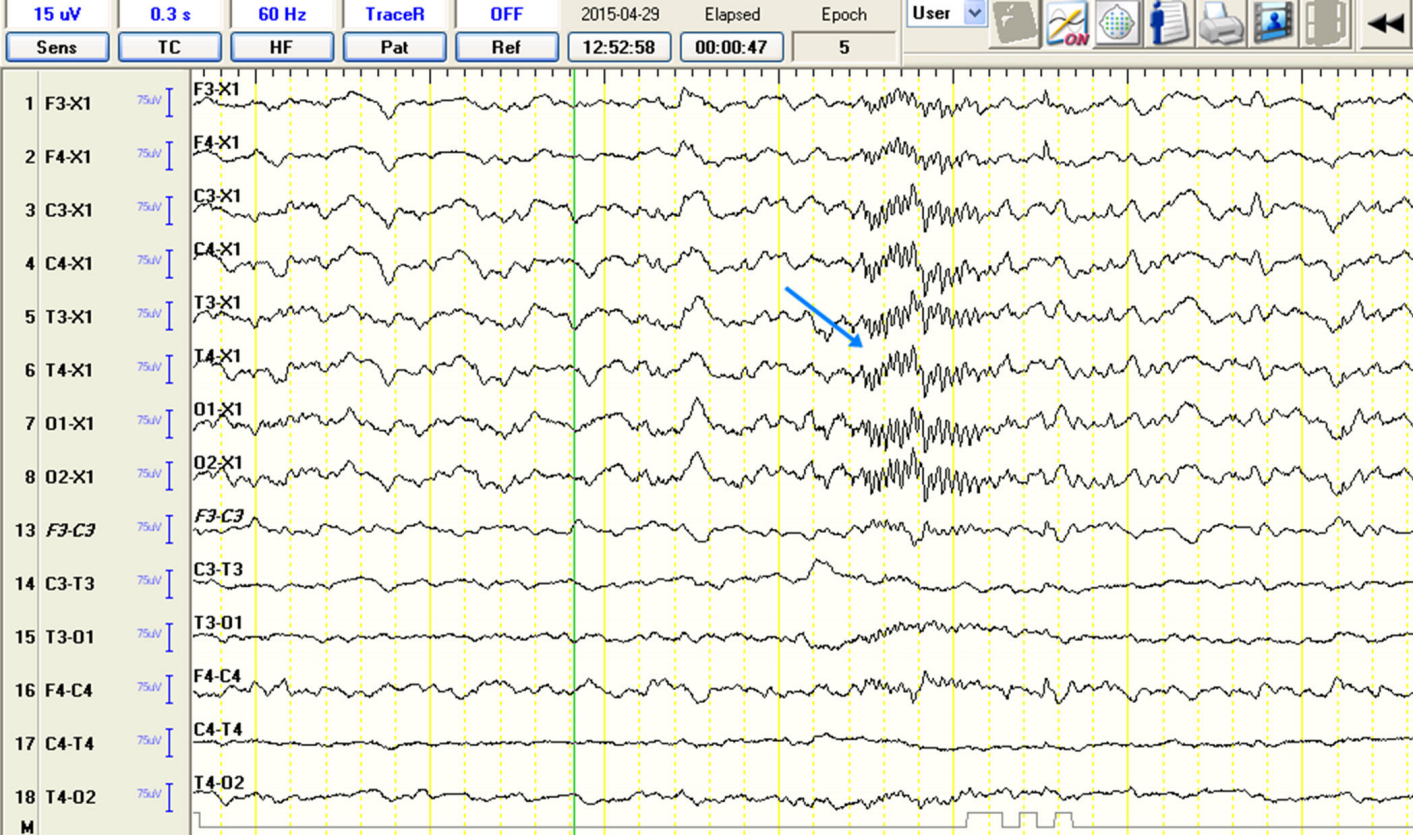
Electroencephalographic recordings from a 3‐year‐old female border collie showing physiological transient—sleep spindle (blue arrow).

Group C, IE‐S, and Phase 1 in group IE‐SA were characterized by HVLF BGAs, mostly comprising theta and delta activity (Figure 3). Physiological SITs, mostly comprising sleep spindles (Figure 5), were most often identified during the first 5 minutes of the recording. In group IE‐SA, Phase 2 was characterized by gradual changes in BGA, with delta and theta band reductions (from HVLF BGA) (Figure 3) in favor of theta and alpha waves (to LVHF BGA) (Figure 4). The mean time of change from HVLF to LVHF BGA was 7 minutes 20 seconds (median, 8 minutes, 16 seconds). By the 10th minute after atipamezole administration, the BGA consisted of alpha (closed eyes) and beta (open eyes) activity in all dogs belonging to the IE‐SA group. Most of the dogs remained or were kept in sternal recumbency after sedation reversal that enabled a 30 minutes EEG acquisition.

A readable EEG recording was obtained in 100% (6/6) cases in groups C and 83% (5/6) cases in groups IE‐S and IE‐SA. The recordings of 16.7% (1/6) cases in that groups were strongly impacted with muscular movement artifacts. Epileptiform discharges in the recording were found in 16.7% (n = 1/6) of the dogs in group C, 66.7% (n = 4/6) of the dogs in IE‐S group, and 83.3% (n = 5/6) of the dogs in IE‐SA group. A total number of 6 (n = 6) EDs were recorded in group C, 88 (n = 88) in group IE‐S, and 185 (n = 185) in group IE‐SA. The EDs consisted of spikes, polyspikes, and sharp waves and were observed in the C (6/0/0), IE‐S (67/12/10), and IE‐SA (200/39/15) groups (Table 2). A greater number of EDs were observed in animals subjected to the “sedation‐awakening” recording protocol (IE‐SA) in comparison to the standard “sedation” protocol (IE‐S). A wide range of artifacts associated with awakening of the dogs was visible. Because of their large number and lack of usefulness for the purpose of this study, they were not quantified.

**TABLE 2:** Types of EDs in control, IE‐S and IE‐SA groups.

		Phase 1			Phase 2		
	Patient no./EDs	Spikes	Polispikes	Sharp‐waves	Spikes	Polispikes	Sharp‐waves
**Control Group**	**1**	3	0	0	3	0	0
	**2**	0	0	0	0	0	0
	**3**	0	0	0	0	0	0
	**4**	0	0	0	0	0	0
	**5**	0	0	0	0	0	0
	**6**	0	0	0	0	0	0
**IE‐S group**	**1**	10	1	1	8	2	2
	**2**	10	1	1	10	2	2
	**3**	9	1	0	5	2	1
	**4**	11	1	0	4	2	3
	**5**	0	0	0	0	0	0
	**6**	‐	‐	‐	‐	‐	‐
**IE‐SA group**	**1**	15	2	1	30	9	4
	**2**	14	1	1	34	9	4
	**3**	12	1	0	24	7	3
	**4**	12	1	0	27	6	2
	**5**	11	0	0	21	3	2
	**6**	‐	‐	‐	‐	‐	‐

Abbreviation: EDs, epileptiform discharges.

### 3.2 Results of the statistical analysis

#### 3.2.1 Spikes

**FIGURE 6:**
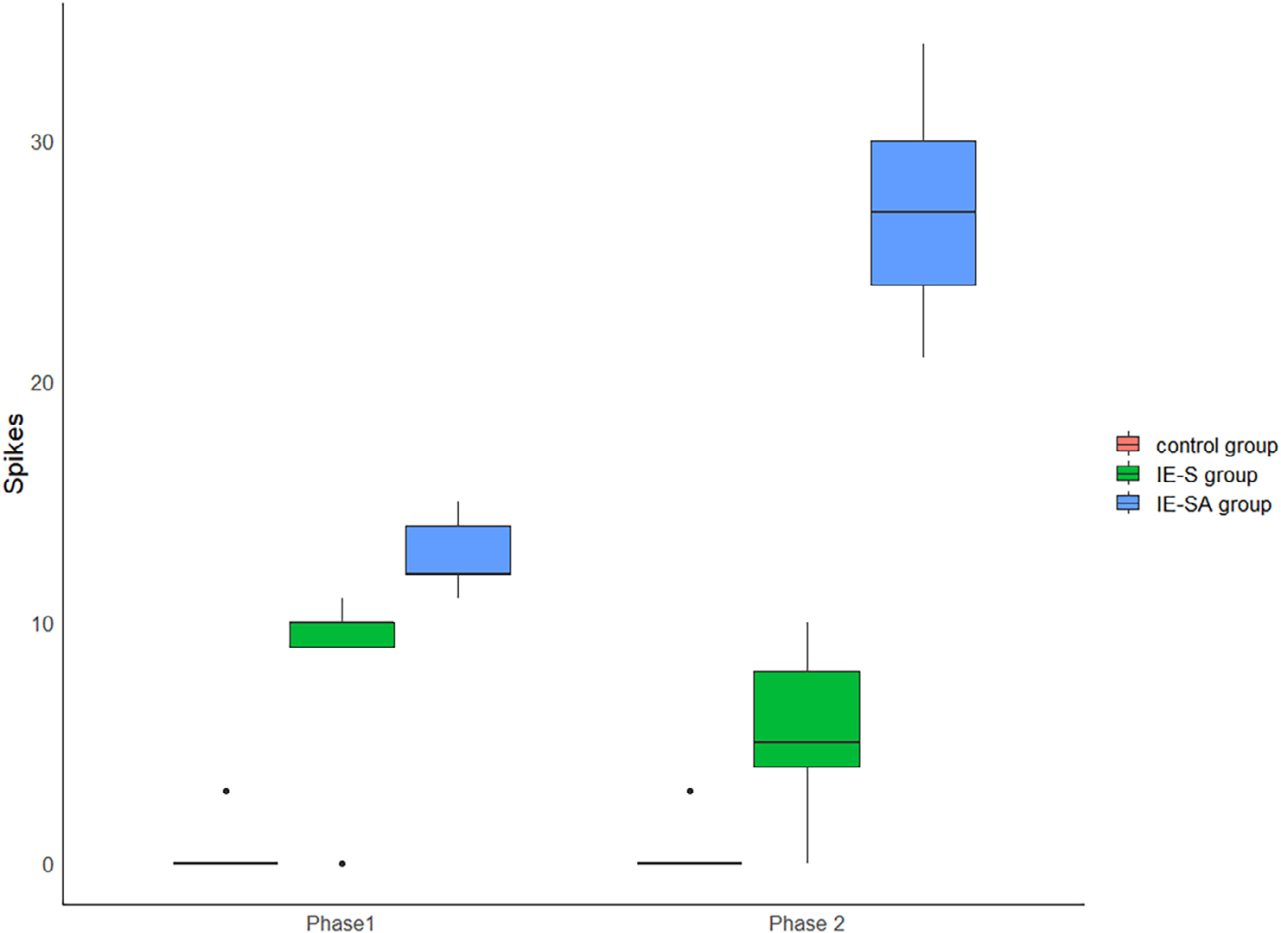
Boxplots comparing EDs (spikes) among the groups. EDs, epileptiform discharges.

Phase 1 showed a statistically significant difference in the number of spikes between groups IE‐S and C (*P* = .031), and IE‐SA and C (*P* = .016) and IE‐S and IE‐SA (*P* = .031). In the second phase, a statistically significant difference in the number of discharges between the IE‐S and C (*P* = .028), IE‐SA and C (*P* = .016), and IE‐S and IE‐SA (*P* = .016) groups was highlighted. A significantly higher number of discharges was observed in the IE‐SA group in both the first and second measurements, the number of discharges in this group increased significantly between measurements (*P* = .011), a slight decrease was observed in the IE‐S group, but it was not statistically significant. The results of the analysis of variance confirmed the presence of a significant interaction between the effect of group and the effect of time—group membership determined the change over time in the outcome (Figure 6).

#### 3.2.2 Polyspikes

**FIGURE 7:**
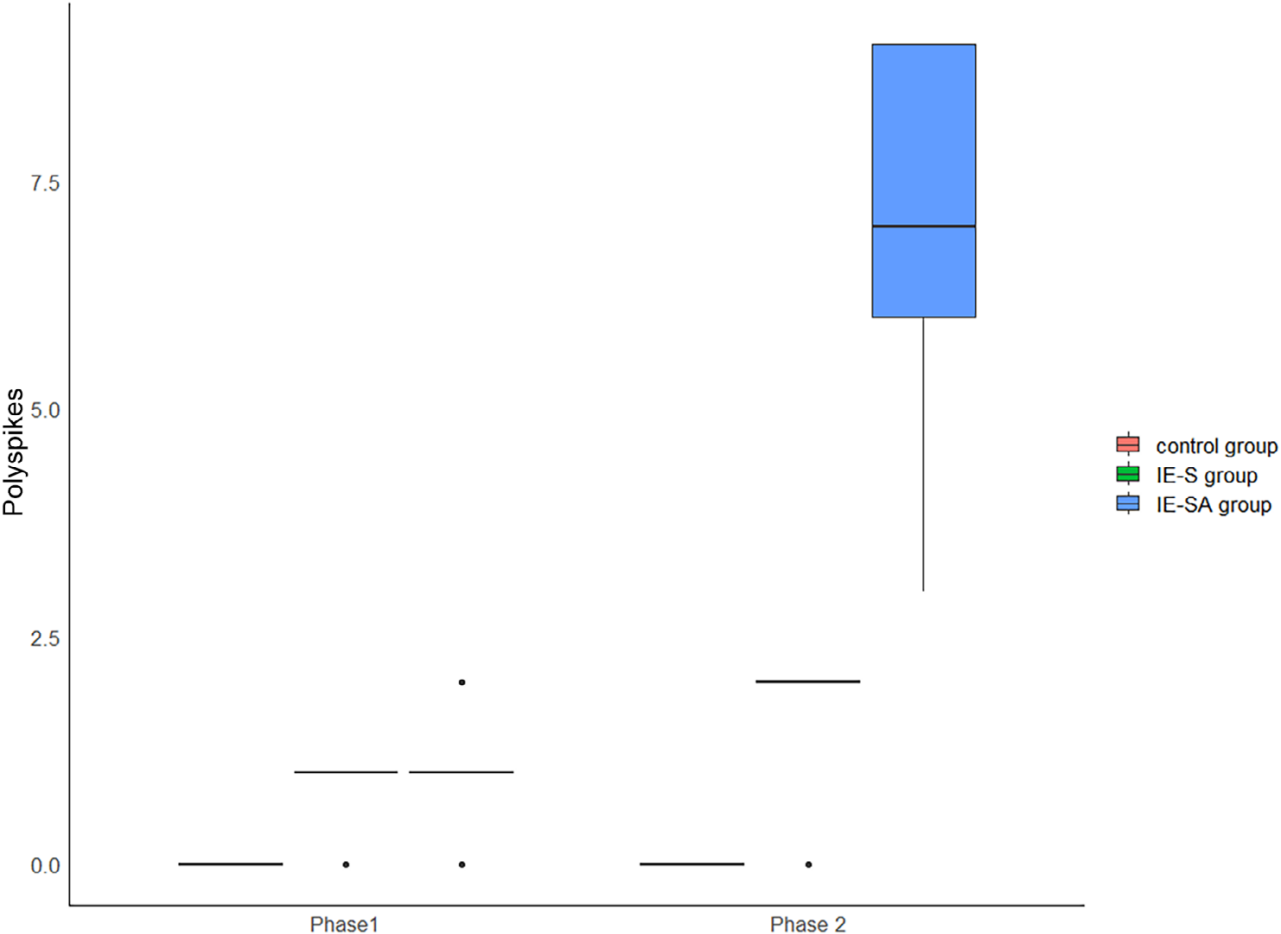
Boxplots comparing EDs (polyspikes) among the groups. EDs, epileptiform discharges.

There were no significant differences in the number of polyspikes between the IE‐S and IE‐SA groups in the first measurement, but both study groups had a significantly higher number than the control (IE‐S and C *P* = .036, IE‐SA and C *P* = .036). The second phase showed a statistically significant difference in the number of discharges among IE‐S and C (*P* = .019), IE‐SA and C (*P* = .011), and IE‐S and IE‐SA (*P* = .019) groups. The number of discharges in the IE‐SA group increased significantly at the second time point (*P* = 0.011). The results of the analysis of variance confirmed the presence of a significant interaction between the effect of group and the effect of time—group membership determined the change in outcome between time points (Figure 7).

#### 3.2.3 Sharp waves

In Phase 1, no statistically significant difference was observed between the number of sharp waves between groups. Phase 2 highlighted a statistically significant difference in the number of discharges between IE‐S and C (*P* = .029), IE‐SA and C (*P* = .011) groups. The number of discharges in the IE‐SA group significantly increased between measurements (*P* = .01), and an increase in the number of sharp wave discharges was observed in the IE‐S group, but it was not statistically significant. The results of the analysis of variance confirmed the presence of a significant interaction between the group effect and the effect of time—group membership conditioned the change in outcome over time.

## 4 DISCUSSION

In the present study, a significant increase in the number of EDs (spikes, polyspikes, sharp waves) in animals subjected to the “sedation‐awakening” protocol was seen in Phase 2, whereas no statistically significant increase in the number of discharges was observed in sedated animals. Analysis of EEG recordings from the 3 groups of dogs revealed significant changes in biological brain activity between the sleep and awake phases. In addition, differences were observed in the EEG records of dogs diagnosed with epilepsy. These differences were in terms of both frequency and characteristics of the discharges. A significantly higher number of EMG artifacts were observed in the IE‐SA group, than in the other groups. When choosing a protocol, it is necessary to take the following factors into consideration: EEG study duration, in terms of the logistics of the study itself and the time required to interpret the study. When sedative is used, the mode of action of sedative and antiepileptic drugs and their effect on the EEG study, along with the experience of the person assessing the findings.

Seizure disorders in dogs are more common than in humans, and affect approximately 0.5% to 5.7% of the general population.^1^, ^19^, ^20^, ^21^, ^22^, ^23^ Therefore, the EEG examination of dogs is potentially of great value. In humans, EEG is the standard diagnostic and monitoring technique used for seizure disorders.^18^, ^24^, ^25^ Electroencephalography acquisition registers fine electrical activity at a specific frequency (0.5‐50 Hz) and amplitude (1‐100 mV), which corresponds to the cortical activity.^8^, ^26^ Therefore, it is highly sensitive to any external electrical activity that causes artifacts, including ocular movements, muscular activity, body movements, cardiac elective activity, and external activity of electric devices.^27^, ^28^ Performing EEG in animals carries additional difficulties as dogs do not maintain a stationary position. To avoid this problem, most reported EEG acquisitions in dogs have been performed using various sedation protocols or in combination with myorelaxants.^11^, ^27^, ^29^, ^30^, ^31^, ^32^ This allowed the reduction of muscle artifacts to avoid misinterpretation of epileptiform spike activity. The influence of sedatives on EEG recordings includes slowing of BGA and appearance of different sleep physiological transients, including sleep spindles and K‐complexes.^33^ Both EEG features (BGA and SITs) have to be properly interpreted by the examiner to differentiate them from pathological BGA (eg, in metabolic encephalopathy)^34^ and SITs (eg, in EDs).^3^, ^29^ If myorelaxants are used, there is a need for animal intubation that requires deep sedation with drugs such as propofol.^29^ In turn, deep sedation might result in lower detection of EDs.^31^, ^35^ Moreover, the use of propofol for EEG acquisition is controversial as it could induce EDs or ED‐like transients.^36^

Use of medetomidine has sufficient restraining effect that enabled EEG recordings and acceptable muscle artifact reduction.^32^, ^33^ Medetomidine has a high affinity for alpha‐2 adrenergic receptors in comparison to other alpha‐2 mimetics.^17^ Unlike the receptors of other anesthetics, alpha‐2 receptors are located in the cerebrum. Therefore, alpha‐2 mimetics do not disturb the cognitive function and induce a state similar to physiological nonrapid eye movement (NREM) sleep phase.^17^, ^33^ Sedation with medetomidine allows for 30 minutes EEG recording in dogs. According to the American Electroencephalographic Society guidelines, a 20 minutes recording is the minimal period required for satisfactory EEG recording.^5^ The only possible disadvantage is that BGA and SIT in NREM sleep EEG recordings induced by medetomidine might potentially lead to the misinterpretation of disseminated encephalopathies.^37^ In such cases (eg, metabolic, degenerative diseases), BGA can be characterized by HVLF discharges because of general BGA slowing, which is an activity similar to that induced by sedation because of increased activity of delta and theta rhythms.^15^, ^34^, ^37^

Standard superficial cup EEG electrodes, because of the associated high number of movement artifacts, might have limited practical application in routine ambulatory EEG in veterinary medicine.^12^, ^13^, ^14^ Therefore, in this study the use of needle electrodes such as SNE and SWE have been proposed.^12^, ^15^ Electrode placement usually requires tranquilization of the dogs; therefore, various anesthetic protocols for EEG recordings have been described. The detection rate of EDs in dogs with these protocols ranges from 12.5%^31^ to 20%‐29%,^27^, ^33^ 55%‐65%,^11^, ^38^ up to 86%‐100%,^39^ and appears to be sedation‐ and montage‐dependent. Interestingly, sleep can facilitate epileptic activity, and seizures tend to occur during specific states of sleep.^40^ This could be interpreted as both facilitating and inhibitory agent for seizure detection. A previous study on a large cohort of dogs (n = 125) with seizures of different origins, EEG recordings were performed using medetomidine sedation; and the ED detection rate was found to be 47% in normal dogs, 88.9% in the group with metabolic seizures, 77.7% in dogs with seizures caused by intracranial lesions, and 61.4% in the group with idiopathic epilepsy.^15^ A study of EEG recordings in unsedated dogs aimed to investigate and determine the etiology of events to differentiate behavioral seizures from epileptic seizures, but did not assess the detection rate for EDs. In this study, 31% dogs required sedation to enable electrode placement.^13^ Sedation, general anesthesia (GA), and concurrent antiseizure drugs (ASD) administration were not identified as confounding factors for decreased ambulatory wireless video electroencephalography (AEEG) diagnostic capability.^41^ In this study, ambulatory EEG was diagnostic in 60.2% of dogs, including 49% of the sedation/GA dogs and 68% of dogs that received neither. The AEEG was diagnostic in 51% dogs with at least 1 ASD and 66% untreated dogs. No difference was found in terms of time to first abnormality among the sedation/GA, ASD‐treated, or untreated dogs. Ninety‐five percent of dogs demonstrated at least 1 abnormality within 277 minutes. The duration of recording should be >4 hours to increase the chance of recording an episode of interest or EDs on the AEEG.^41^

Electroencephalography examination on a fully awake dog is associated with several technical obstacles, including difficulty in proper electrode placement on the dog's scalp and gaining proper resistance (<10 kΩ). Hence, this study proposes a protocol that was combined with medetomidine sedation, which was reversed after 5 minutes with atipamezole. This “sedation‐awakening” protocol allows proper electrode placement, measurement or improvement of resistance if needed, and the preparation for accurate technical recording. This protocol allows the observation of cortical activity during the sedation‐recovery and awakening phases. In all cases, this protocol allowed EEG recording for the desired 30 minutes.

There are no reports or pharmacokinetic premises indicating atipamezole effect on the occurrence of seizure attacks and thereby on the occurrence of EDs in EEG recordings.^42^, ^43^, ^44^, ^45^ Atipamezole is routinely administered to healthy dogs that require short‐term sedation, muscle relaxation, and analgesia.

**FIGURE 8:**
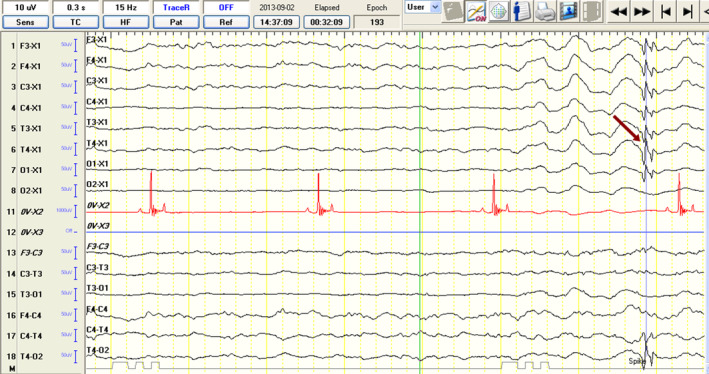
Electroencephalographic recordings from a 3‐year‐old female border collie showing spike activity (red arrow) from the T4 lead and HVLF BGA. Discharge localization is defined based on the highest amplitude in the reference montage and reversed polarity in the bipolar montage recording. BGA, background activity; HVLF, high voltage, low frequency.

**FIGURE 9:**
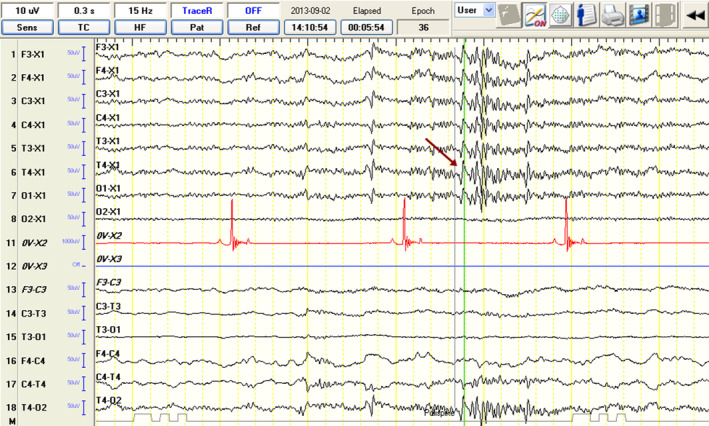
Electroencephalographic recordings from a 3‐year‐old female border collie showing polyspike activity (red arrow) from the T4 lead and LVHF BGA. Discharge localization is defined based on the highest amplitude in the reference montage and reversed polarity in the bipolar montage recording. BGA, background activity; LVHF, low voltage, high frequency.

Although the examination room was dim and quiet, and sedation recovery in the IE‐SA group was slow and calm, a significantly higher number of EMG artifacts were observed (Figure 2). Eye movements, muscle activity, and body movements of each dog can cause artifacts resembling pathological brain activity. Therefore, particular bursts can be differentiated more feasibly as pathological based on analysis of the images from the camcorder (Figure 2), which recorded dog positioning during the entire examination. Movement and muscle artifacts can significantly increase the number of misinterpreted SITs.^26^ In this study, EDs (Figures 8 and 9) were identified based on the International League Against Epilepsy (ILAE) recommendations.^3^, ^18^, ^24^

**FIGURE 10:**
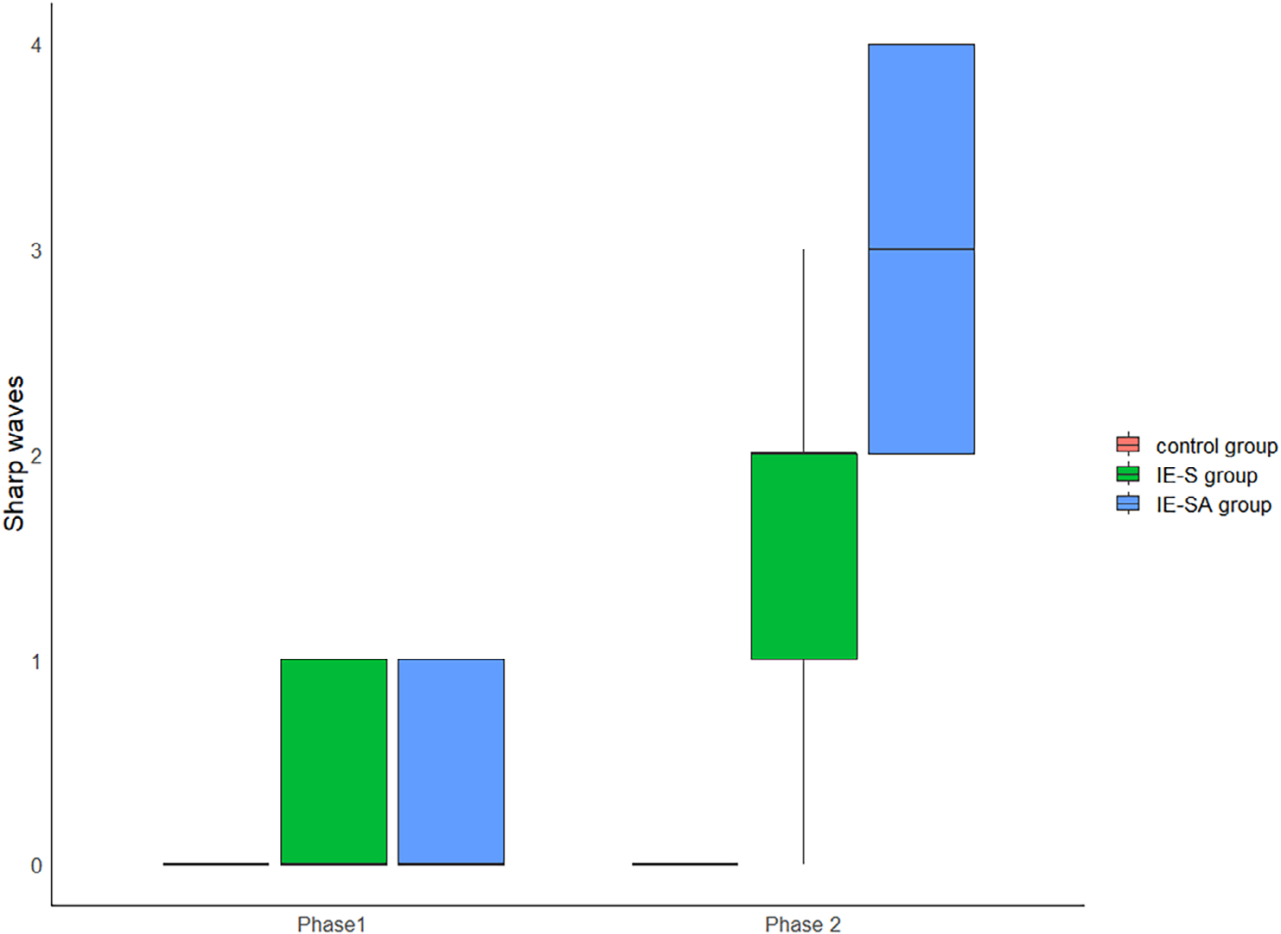
Boxplots comparing EDs (sharp waves) among the groups. EDs, epileptiform discharges.

The change in BGA from HVLF to LVHF (Figure 10) in the 10th minute of recording and approximately 5 minutes after administering atipamezole injection in the IE‐SA group was related to the waking phase of the animals. A similar EEG pattern is observed during sleep, which consists of 2 phases: NREM (high tension, slow activity), which consists of 4 substages; and REM (rapid‐eye movement) (low tension, quick activity). Sedation with medetomidine was found to exhibit all NREM phase characteristics. Medetomidine itself does not induce abnormal SITs of EDs.^32^, ^46^ Therefore, 1 might conclude that all abnormal findings obtained during medetomidine sedation could be valuable. During medetomidine sedation, physiological SITs of sleep spindles and K‐complexes might be present during the NREM sleep phase.^35^ Care should be taken to differentiate between transient and epileptic SITs. Different animal reactions to atipamezole must be considered as not all dogs awoke at the same time as the BGA were present from 5 to 10 minutes after the administration of atipamezole.

The lack of EDs during interictal EEG recordings does not rule out epilepsy.^25^ Analysis of EEG recordings from the 3 groups of dogs revealed significant changes in biological brain activity between the sleep and awake phases. In addition, differences were observed in the EEG records of dogs diagnosed with epilepsy. These differences were observed in terms of both frequency and characteristics of the discharges. However, this protocol has several limitations that need consideration. An awake animal is likely to move and is also more sensitive to the surrounding stimuli. As a result, these recordings are likely to reflect the bioelectrical activity and artifacts. The temperament of animals should also be considered. If a dog is extremely aggressive or energetic, it is impossible to obtain well‐defined recordings in the awake state. Finally, the conditions under which this examination was conducted required absolute isolation without the participation of a third party. The recording room was quiet and shaded to reduce artifacts.

## 5 CONCLUSIONS

An accurate definition of the phenotype of an epileptic disorder must include EEG data obtained and interpreted by a trained neurologist specialized in the study of epileptic disorders, especially for those with suspected genetic etiology.^46^ In conclusion, this protocol is not recommended if another medical procedure is to be conducted on the same day as EEG recording under GA. This protocol could be of great value for repeated EEG recordings (as advised by the ILAE) during control appointments or for monitoring epilepsy treatment. The use of a “sedation‐awakening” protocol can be a valuable alternative to confirm the neurological origin of seizures.

## CONFLICT OF INTEREST DECLARATION

Authors declare no conflict of interest.

## OFF‐LABEL ANTIMICROBIAL DECLARATION

Authors declare no off‐label use of antimicrobials.

## INSTITUTIONAL ANIMAL CARE AND USE COMMITTEE (IACUC) OR OTHER APPROVAL DECLARATION

Authors declare no IACUC or other approval was needed.

## HUMAN ETHICS APPROVAL DECLARATION

Authors declare human ethics approval was not needed for this study.
